# The association between body image perception and metabolic syndrome in normal-weight adults

**DOI:** 10.1371/journal.pone.0284294

**Published:** 2023-04-10

**Authors:** Jieun Shin, Sungjung Kwak

**Affiliations:** 1 Department of Biomedical Informatics, College of medicine, Konyang University, Daejeon, Republic of Korea; 2 Department of Nursing, Howon University, Gunsan, Republic of Korea; Endocrinology and Metabolism Population Sciences Institute, Tehran University of Medical Sciences, ISLAMIC REPUBLIC OF IRAN

## Abstract

**Aim:**

This study analyzed the association between metabolic syndrome and its components according to body image perception in normal-weight adults.

**Background:**

Management of chronic diseases that are the main cause of death in Korea is essential. The representative cause of the increase in these chronic diseases is metabolic syndrome, and preventing it is essential for chronic disease management.

**Methods:**

For data analysis, this study used data from Korea National Health and Nutritional Examination Survey (KNHANES-VII), 2016–2019. A total of 6479 survey respondents with normal BMI, aged 19 to 64 years old, were finally selected for analysis. Analysis was performed separately for men and women because gender-dependent differences were observed in the self-perceived body image types (underestimation, congruence, and overestimation groups). The incidence and risk of metabolic syndrome-related indicators according to body image perception were analyzed.

**Results:**

As a result, it was found that men tending toward underestimation had lower prevalence and risk of metabolic syndrome and women tending toward overestimation had higher prevalence and risk of metabolic syndrome.

**Conclusion:**

This suggests that even individuals with the same BMI can have effects on the prevalence and risk of metabolic syndrome depending on their body image perception. This allows the conclusion that subjective body image perception can function as a supplementary predictor of metabolic syndrome.

## Introduction

According to 2019 Statistics Korea data, chronic diseases accounted for 79.7% of all deaths in South Korea, with 8 of the 10 leading causes of death being chronic diseases [1]. The burden of chronic diseases is expected to keep increasing due to the increase in the elderly population, with the total economic cost of chronic diseases in the period from 2010 to 2030 projected to reach US$ 1 trillion [2]. In response, the WHO set a goal to reduce the mortality due to cardiovascular disease, cancer, diabetes, or chronic respiratory disease by 25% by 2025 [3], and metabolic syndrome was confirmed to be the major cause of the increase in cardiovascular disease and diabetes [4].

Metabolic syndrome is a cluster of three or more of five risk factors, namely diabetes, hypertension, hypercholesterolemia, hypertriglyceridemia, and obesity [5]. According to the National Health Screening Statistical Yearbook 2020, 69.8% of those screened had one or more metabolic syndrome risk factors [5]. As such, metabolic syndrome has increased rapidly in South Korea over the past few decades, and metabolic syndrome prevention is an essential part of chronic disease management.

Obesity has been identified as a major risk factor for metabolic syndrome, and body mass index (BMI) is widely used as an indirect predictor for evaluating the risk of metabolic syndrome [6]. However, BMI may vary by race and lifestyle, and even individuals with the same BMI may have different risk of metabolic syndrome depending on various factors such as smoking and drinking [7].

Moreover, normal-weight individuals also present metabolic disturbances characteristic of obese individuals, which is termed “metabolically obese but normal weight” (MONW) [8]. MONW individuals are at higher risk of developing type 2 diabetes mellitus and cardiovascular disease [9, 10], but their low BMI values used to mislead both physicians and patients to underestimate their risk of contracting the disease [11]. This made it necessary to find additional predictors to compensate for this diagnostic inaccuracy, and body image perception has recently garnered great attention [12].

Although the correlation between metabolic syndrome and obesity has been investigated in previous studies, individual body perception of self-image has mainly studied to examine its association or correlation with BMI, and with the subjects of these studies limited to adolescents, there is a problem of generalizing research findings to adults.

Therefore, this study aims to provide basic data for setting up strategies and countermeasures to prevent and control metabolic syndrome and further reduce the incidence of chronic diseases by evaluating the frequency and risk of metabolic syndrome attributable to subjective body image perception among normal-weight adults.

## Methods

### Participants

For data analysis, this study used data from the Seventh and eighth Korea National Health and Nutritional Examination Survey (KNHANES-VII), 2016–2019. Of the 32,379 survey respondents in total, 7,330 individuals met the selection criteria of (i) adults aged 19 to 64 years (n = 19,304) and (ii) normal BMI. After applying exclusion criteria, namely (i) individuals with reduced mobility (n = 288), (ii) pregnant or breastfeeding women (n = 92), (iii) cancer patients (n = 294), and (iv) individuals under dietary control (n = 279), 6,479 survey respondents were finally selected for analysis.

### Instruments

#### BMI measurement and classification criteria for body image perception

BMI was calculated by dividing the measured body weight (kg) by the square of the height (m) (kg/m2). Body measurements were directly measured by nurses who completed 2 to 4 weeks of education and practice, height was measured using n extensometer (SECA 274, SECA, Germany), and weight was measured using a scale (Inbody 970, Biospace, Korea). Then the subjects falling into the category of “right weight” (normal weight, BMI 18.5–22.9 kg/m2) were selected based on the cutoff values set in the World Health Organization Asia-Pacific guidelines and the Korean Society for the Study of Obesity guidelines [13].

Subjective body image perception was divided into three groups: underestimation group (very thin and slightly thin in the KNHANES classification), congruence group (normal weight), and overestimation group (slightly obese and very obese).

### Diagnostic criteria for metabolic syndrome

Based on the modified NCEP-ATP III criteria for diagnosing metabolic syndrome, this study defined metabolic syndrome as the presence of three or more of the following criteria: abdominal obesity, impaired fasting glucose, hypertriglyceridemia, dyslipidemia, and hypertension, of which the cut off points are as follows:

1. Waist circumference (WC): ≥ 90cm in men, ≥ 85cm in women
2. Hypertension: blood pressure ≥ 130/85 mmHg or under medication
3. Hypertriglyceridemia: TG ≥ 150 mg/dL
4. Impaired fasting glucose: Fasting glucose ≥ 100 mg/dL or during drug treatment
5. Dyslipidemia: fasting HDL-cholesterol < 40 mg/dL in men, < 50 mg/dL in women

### Data analyses

Statistical analysis was performed using SAS ver. 9.4 (SAS Institute Inc, Cary, NC, USA), and complex sample analysis was performed using the weights specified in the KNHANES raw data utilization guidelines released by the Korea Disease Control and Prevention Agency.

Analysis was performed separately for men and women because gender-dependent differences were observed in the self-perceived body image types (underestimation, congruence, and overestimation groups).

First, cross-analysis was performed to determine the distribution of the self-perceived body image types according to the subject characteristics. Next, GLM analysis was performed on the mean values of metabolic syndrome-related indicators according to the self-perceived body image types, followed by cross-analysis in order to compare the incidence rates across the components of metabolic syndrome depending on the reference values. Finally, logistic regression analysis was performed to compare the odds ratio (OR) for metabolic syndrome between the self-perceived body image types.

### Ethical considerations

This study represents a secondary analysis of national survey data available through the Korea National Health and Nutrition Examination Survey website and was conducted after receiving approval from the institutional review board of K University (KYU 2022-08-018). Because the raw data analyzed for this study contain no personally identifiable information, anonymity and confidentiality were guaranteed.

## Results

### Participants’ general characteristics

Whereas 58.1% (n = 3,908) of normal-weight adults (n = 6,479) perceived their body image as normal weight, 26.5% (n = 1,554) and 15.4%(n = 1,017) were found to have distorted self-images of underweight and overweight, respectively.

**Fig 1:**
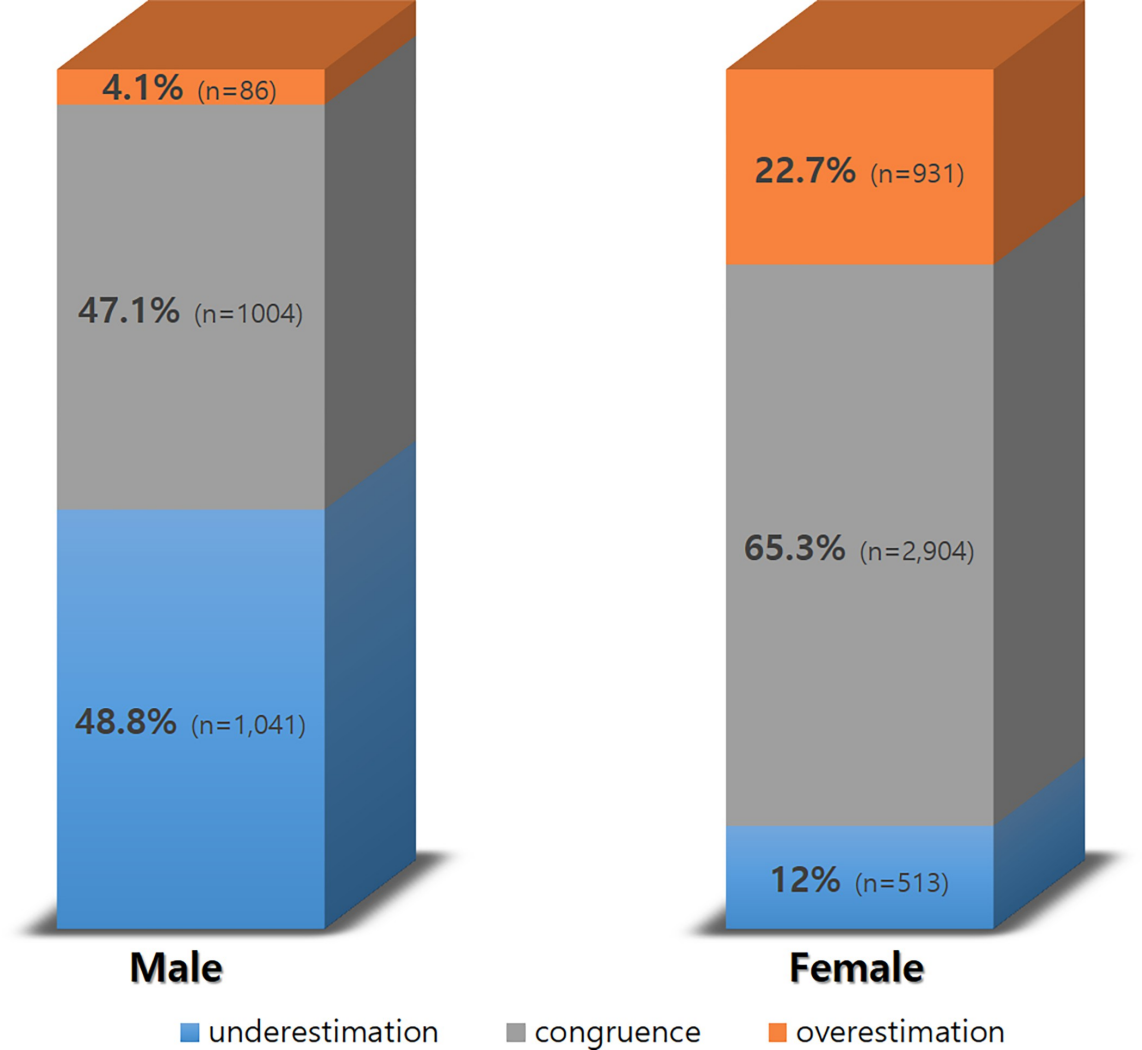
Differences in body image perception according to gender.

The gender-dependent differences in body image perception among normal-weight adults were found to be statistically significant (p < .001). The largest proportion of men (48.8%) had a distorted perception of self-image as underweight, and the smallest proportion of men (4.1%) had a distorted perception of self-image as overweight. In contrast, the largest proportion of women (65.3%) had a congruent perception of self-image as normal weight, with a 36%p lower of underweight perception a 18%p higher overweight perception than men (Fig 1).

Analysis of the differences in body image perception according to subjects’ general characteristics revealed that men showed statistically significant differences in body image perception depending on the household income level and whether involved in economic activities or not, and women depending on age and education level.

In adult men, the proportion of congruent body image perception increased as the household income rising from Q2 to Q4 (Q2: 43.3%, Q3: 46.2%, Q4: 49.7%), and those involved in economic activities showed about 4%p higher proportion of a congruent body image perception than those not involved in economic activities (48.6% vs 44.8%).

In adult women, as the age advanced, the proportion of congruence group increased, with a downward tendency of overestimation group. In terms of education level, which is highly correlated with age group, those who completed middle school had the highest proportion of congruent body image perception, with a higher education level associated with a lower proportion of underestimation and a higher proportion of overestimation (Table 1).

**Table 1:** Participants’ general characteristics (N = 6,479).

		Male					Female				
		Under estimation	congruence	Over estimation	χ^2^	p	Under estimation	congruence	Over estimation	χ^2^	p
Age	-29	263(50)	243(46.2)	24(3.7)	14.453	.071	91(10.5)	546(62.6)	225(26.8)	51.607	< .001
	30–39	228(53.7)	182(40.5)	22(5.8)			102(11.1)	666(62.4)	268(26.5)		
	40–49	221(45.4)	239(50.7)	16(3.9)			127(11.8)	768(65)	258(23.2)		
	50–59	214(45.6)	233(51.5)	15(2.8)			139(14.8)	670(71.3)	132(14)		
	60+	115(48.9)	107(45.5)	9(5.6)			54(14.1)	254(70.1)	48(15.8)		
Marital status	Sigle	384(50.2)	350(45.1)	38(4.7)	3.255	.516	106(10.9)	647(63.4)	253(25.7)	7.153	.128
	married_Yes	613(47.9)	615(48.4)	45(3.7)			362(12.3)	2028(66)	624(21.7)		
	married_No	44(48.1)	39(49.7)	2(2.2)			45(13.7)	227(66.3)	54(20.1)		
Household income	Q1	103(45.5)	105(50.2)	11(4.2)	14.205	.027	45(13.6)	212(61.3)	72(25.1)	4.034	.672
	Q2	259(52.4)	215(43.3)	18(4.3)			113(12.9)	620(63.5)	208(23.7)		
	Q3	347(51.5)	313(46.2)	16(2.3)			159(11.4)	882(66.1)	283(22.4)		
	Q4	330(44.7)	370(49.7)	40(5.6)			196(11.8)	1182(66.5)	363(21.7)		
Economic activities	Yes	780(48.9)	771(48.6)	44(2.5)	6.609	.037	295(11.9)	1774(66.3)	542(21.8)	0.527	.768
	No	203(50.2)	184(44.8)	17(5)			195(12.2)	1024(65)	328(22.8)		
Education level	Elementary	34(44.5)	46(47.8)	6(7.8)	9.382	.153	36(20)	96(64.8)	23(15.2)	22.903	.001
	Middle	80(58.4)	59(39.6)	4(2)			41(17)	194(70.4)	34(12.6)		
	High	429(49.6)	394(47.6)	21(2.7)			164(11.1)	1001(65.6)	319(23.3)		
	University	443(47.9)	460(49.1)	30(3)			249(11.6)	1511(65.6)	493(22.8)		

χ^2^: Rao Scott Chi-square test.

### Body image perception and metabolic syndrome-related indicators

The differences in the prevalence of metabolic syndrome according to body image perception were found to be statistically significant in both men and women. In men, the underestimation group had the lowest prevalence (6.8%), followed by congruence group (12.9%) and the overestimation group (15.4%). In women, the congruence group had the lowest prevalence (4.2%), followed by the underestimation group (5.1%) and the overestimation group (6.7%).

Statistically significant differences were observed in WC for both men and women. Men’s WC increased in the order of underestimation (mean = 76.48cm), congruence (79.38cm), and overestimation (80.60cm). In women as well, WC increased in the order of underestimation (69.12cm), congruence (71.68cm), and overestimation (74.28cm).

TG and HDL showed statistically significant differences according to body image perception in women. TG increased in the order of underestimation group (86.68mg/dl), congruence group (87.59 mg/dl), and overestimation group (94.77 mg/dl). HDL decreased in the order of underestimation (90.38 mg/dl), congruence (59.70 mg/dl), and overestimation (57.91 mg/dl).

Systolic blood pressure (SBP) and diastolic blood pressure (DBP) showed statistically significant differences only in men. The congruence group showed the highest SBP (116.14mmHg), followed by the overestimation group (115.12mmHg) and the underestimation group (114.14mmHg). The congruence group showed the highest DBP (76.94mmHg), followed by the underestimation group (75.45mmHg) and the overestimation group (75.18mmHg).

Glucose showed the lowest value in the underestimation group in men and the congruence group in women, but without reaching statistical significance (Table 2).

**Table 2:** Body image perception and metabolic syndrome related indicators.

		Under estimation	Congruence	Over estimation	Total	F	p-value
Male	WC	76.48±0.17	79.38±0.16	80.60±0.60	78.01±0.13	80.04	< .001
	TG	126.86±5.33	136.40±4.40	133.63±9.39	131.63±3.47	1.03	.357
	HDL	52.45±0.40	51.19±0.43	50.82±1.21	51.79±0.29	2.81	.061
	SBP	114.14±0.43	116.14±0.49	115.08±1.80	115.12±0.33	4.98	.007
	DBP	75.45±0.32	76.94±0.38	75.18±1.34	76.14±0.25	4.74	.009
	Glucose	94.79±0.60	96.75±0.67	95.57±2.12	95.75±0.44	2.34	.098
Female	WC	69.12±0.24	71.68±0.12	74.28±0.20	71.96±0.10	151.69	< .001
	TG	86.68±2.94	87.59±1.28	94.77±2.31	89.11±1.01	3.99	.019
	HDL	60.38±0.70	59.70±0.29	57.91±0.47	59.37±0.24	6.56	.002
	SBP	109.19±0.65	108.98±0.27	108.23±0.48	108.83±0.23	1.10	.333
	DBP	71.82±0.46	72.22±0.20	71.84±0.34	72.08±0.77	0.72	.487
	Glucose	92.19±0.80	91.13±0.24	92.08±0.49	91.47±0.22	2.13	.120

F: GLM(General linear model) analysis.

Among the five risk factors of metabolic syndrome, adult men showed a statistically significant differences in the prevalence rates of TG, HDL, HP, and glucose, and adult women in WC, TG, and HDL. In the metabolic syndrome risk factors, both male and female adults showed a low prevalence in the underestimation group. But, in the case of female adults, the prevalence was low in the case of congruent body type recognition in WC (Table 3).

**Table 3:** Detailed indicators related to metabolic syndrome by group.

		Under estimation	Congruence	Over estimation	Total	χ^2^	p-value
Male	WC	4(0.3)	8(0.8)	3(2.3)	15(0.6)	5.511	.064
	TG	237(21.7)	284(27.9)	21(29)	542(24.9)	9.066	.011
	HDL	205(19.4)	252(24.8)	16(21.4)	473(22.0)	6.055	.048
	HP	238(21.4)	293(28.5)	25(25.6)	556(24.9)	10.998	.004
	Glucose	215(20.3)	261(24.9)	24(30.5)	500(22.9)	6.880	.032
	Metabolic syndrome	75(6.8)	132(12.9)	11(15.4)	218(10.0)	18.379	< .001
Female	WC	7(1)	19(0.6)	22(2.4)	48(1.1)	20.029	< .001
	TG	44(7.1)	256(8.3)	119(12.7)	419(9.2)	15.716	< .001
	HDL	149(27.7)	892(29)	321(34)	1362(30.0)	7.209	.027
	HP	85(14.9)	436(13.5)	126(12.1)	647(13.4)	1.953	.377
	Glucose	64(12.3)	370(12)	138(12.8)	572(12.2)	.355	.838
	Metabolic syndrome	29(5.1)	147(4.2)	67(6.7)	243(4.9)	7.950	.019

χ^2^: Rao Scott Chi-square test.

### Analysis of the risk of metabolic syndrome according to body image perception

The risk of metabolic syndrome was compared using that of the congruence group as the reference value. The comparison led to the findings that adult men’s OR (CI) decreased in the underestimation group (0.49(0.35–0.69)) and adult women’s OR (CI) increased in the overestimation group (1.62(1.17–2.26)).

Among the five components of metabolic syndrome, men showed a statistically significant decrease in the risk of metabolic syndrome in terms of TG, HDL, HP, and glucose in the underestimation group, and women showed a statistically significant increase in the risk of metabolic syndrome in terms of WC, TG, HDL in the overestimation group (Table 4).

**Table 4:** Metabolic syndrome risk by group.

		Underestimation	Congruence	Overestimation
Male	WC	0.409(0.095–1.773)^1)^	1	3.001(0.671–13.409)
	TG	0.716(0.573–0.895)	1	1.051(0.595–1.854)
	HDL	0.732(0.579–0.925)	1	0.829(0.414–1.660)
	HP	0.681(0.538–0.863)	1	0.861(0.497–1.493)
	Glucose	0.767(0.610–0.964)	1	1.325(0.740–2.372)
	Metabolic syndrome	0.490(0.347–0.692)	1	1.236(0.594–2.572)
Female	WC	1.593(0.678–3.917)	1	3.832(1.903–7.719)
	TG	0.841(0.570–1.240)	1	1.604(1.222–2.105)
	HDL	0.937(0.731–1.200)	1	1.259(1.049–1.511)
	HP	1.124(0.834–1.515)	1	0.880(0.683–1.133)
	Glucose	1.033(0.742–1.438)	1	1.076(0.847–1.368)
	Metabolic syndrome	1.228(0.754–1.999)	1	1.623(1.167–2.257)

^1)^ Odds ratio(95% Confidence Interval): Logistic regression analysis.

## Discussion

In Korea, increase in the elderly population came with a rapid increase in the incidence of metabolic syndrome, which is expected to keep increasing in the future [5]. It is thus necessary to set up effective strategies to prevent and control chronic diseases likely to increase under these circumstances.

BMI is widely used as an indirect predictor of metabolic syndrome as an indicator of obesity, a typical cause of metabolic syndrome [6]. However, given the inconsistency of its values depending on various factors involves [7] this study focused on the risk of metabolic syndrome depending on subjective body image perception.

First, in the subjective perception of body image, 58.1% of the respondents had the same body image perception corresponding to their actual body weight, with the remaining 41.9% showing negatively perceived body image by over- or underestimating their own body weight. This result supports the research finding that even though overweight or obesity in Korea is not at a serious level when compared with Western countries, the inconsistency between objectively measured body weight type and subjectively perceived body image in Korean adults surpasses that of their Western counterparts [14].

As regards gender-dependent differences, the largest proportion of men (48.8%) had a distorted perception of self-image as underweight, and the largest proportion of women (65.3%) had a congruent perception of self-image as normal weight. These results suggest that the majority of adult women have body image perception congruent with their actual body weight, whereas adult men tend to have body image perception underestimating their actual body weight. Men’s obesity rate tends to increase to a greater extent than that of women [15], and men had a lower proportion of body image perception congruent with their actual weight although they have a higher proportion of BMI-based overweight than women [16]. In addition, 17–19% of overweight and obese men underestimated their own weight [17], and the likelihood of underestimating their own weight increased by 1.13 times with each increase in BMI class [18]. In contrast, both men and women had the highest rate in correct estimation in a study with adult subjects (≥ 20 years) [13], and men had the highest rate in correct estimation and women in overestimation (56.3%) in another study [19]. In addition, in a meta-analysis study conducted in the United States, more men were found to have an overestimated perception of their weight than women [20]. Although gender has been identified as a determinant of body image perception in the studies conducted so far, there are interstudy differences regarding gender-dependent differences in body image perception (underestimation/congruence/overestimation). It is considered necessary to determine the gender-dependent differences through replication studies to be reflected in the intervention strategy for effective chronic disease prevention.

Subjective body image perception according to the subjects characteristics showed gender differences. Men’s body image perception varied depending on the household income level and whether involved in economic activities or not, and women’s body image perception varied depending on age and education level. Men’s correct estimation rate increased and underestimation rate decreased as household income increased, and their underestimation or overestimation rate increased when they were not involved in economic activities. That is, adult men with a higher household income and economic activities were less likely to have a distorted body image perception. Adult women in their 20s were twice more likely to overestimate their weight than to underestimate, perceiving themselves to weigh more than they actually do, and their correct estimation rate increased as the age advanced. That is, women’s body image perception becomes increasingly positive as they grow older, which is consistent with the results of an Australian study [9]. These results are presumably due to women’s tendency to attach more importance to health and bodily functions as they get older and accept more readily aging-related physical changes [21, 22]. In terms of education level, a symmetrical tendency was observed, whereby those with a lower education level (≤ middle school) tended toward underestimation and those with a higher education level (≥ high school) tended toward overestimation. This result is consistent with the finding of a previous study with adult [19]. It can be seen that overestimate their body image are prevalent among young or educated women who sensitive to their body image in relationships with others and relatively strong craving for the ideal body image.

With respect to metabolic syndrome-related indicators in relation to subjective body image perception, WC-related body image perception showed statistically significant intergroup difference in both men and women, where overestimation group had the highest mean WC value. Men showed intergroup differences in both SBP and DBP, with the underestimation group showing higher SBP and the overestimation group showing higher DBP, but without significant differences from the mean values (115/76mmHg). Women showed intergroup differences in TG and HDL, with the highest TG measured in the overestimation group and the highest HDL measured in the underestimation group. In metabolic syndrome, the higher the WC, TG, SBP, and DBP are, and the lower the HDL is, the higher the risk of developing the disease becomes. From the finding that the overestimation group had higher WC and TG and lower HDL, it can be inferred that the risk of metabolic syndrome increases in cases where normal-weight adults have a distorted perception of weighing more than they actually do.

Analysis of the prevalence of metabolic syndrome and its components by group led to the following findings: First, in the prevalence of metabolic syndrome, statistically significant intergroup differences were observed in both men and women, with the overestimation group showing the highest prevalence of metabolic syndrome. Second, in the components of metabolic syndrome, significant intergroup differences were observed in TG, HDL, HP, and glucose in men, with the underestimation group showing the lowest prevalence. In women, there were significant differences in WC, TG, and HDL, with the overestimation group showing the highest prevalence. These results suggest that distorted body image perception is associated with metabolic syndrome, and in the case of men, those underestimating their body weight tend to have lower prevalence of metabolic syndrome as well as its components, and those overestimating their body weight tend to have higher prevalence of metabolic syndrome. In the case of women, those overestimating their body weight tend to have higher prevalence of metabolic syndrome as well as its components. These findings are consistent with those of a previous study [23] that the more obese the body image perception, the higher the risk of developing metabolic syndrome along with the risk profiles of HP, glucose, TG, WC, and HDL. The more people perceive their body type as being obese, the lower the rate of practicing healthy behaviors to prevent chronic diseases such as unhealthy eating habits and lack of exercise [22]. For this reason, it can be inferred that even with the same BMI and body size, depending on body type recognition, the person may react as overweight compared to the body size. Meanwhile, components of metabolic syndrome showed gender-dependent differences according to body image perception, which highlights the need to conduct replication studies to analyze the factors influencing metabolic syndrome related to body image perception and propose appropriate intervention strategies based on the research findings.

Lastly, comparison of the risk of metabolic syndrome using the congruence group as the reference group, the male subjects’ underestimation group had lower risk of metabolic syndrome and its components (TG, HDL, HP, and glucose) and the female subjects’ overestimation group had higher risk metabolic syndrome and its components (WC, TG, and HDL). These results are supported by the finding of a study that even after adjusting for BMI, the greater the discrepancy between actual weight and self-perceived weight, the higher the likelihood of developing metabolic syndrome [24], and a study which demonstrated that individuals who maintained a higher level of weight discrepancy had 200% higher risk of diabetes that those who maintained a lower level of weight discrepancy [25]. As examined above, actual/perceived weight discrepancy appears to affect the risk of disease irrespective of obesity [24]. If weight discrepancy is chronically maintained, excessive stress or negative effects may be experienced, which is thought to have occurred by inducing numerous physiological changes (e.g., immunity, metabolism, and inflammation) and behavioral changes that potentially increase the risk of chronic disease [26].

Looking at the studies conducted so far, they mainly targeted women and adolescents, and most of the studies were related to the effects of overestimation. Our study compared not only overestimation but also overestimated, congruent, and underestimated body image perceptions in both male and female adults. most importantly, in the case of men, when underestimating their body weight, it was found that the risk of metabolic syndrome and related sub-regions was rather reduced. Although our research results have limited generalizability, this study is significant as the first attempt of its kind, and it is necessary to verify the impact of underestimation through replication studies.

## Conclusions

In view of the results of this study, it is considered worthwhile to use body image perception, along with BMI, as a predictor of the risk of chronic diseases such as metabolic syndrome. Previous studies have demonstrated that the more overweight the body is perceived, the higher the risk of metabolic syndrome becomes. In this study, we compared the cases of body weight overestimation, congruent estimation, and underestimation and analyzed the prevalence and risk of metabolic syndrome and its components. As a result, it was found that men tending toward underestimation had lower prevalence and risk of metabolic syndrome and women tending toward overestimation had higher prevalence and risk of metabolic syndrome. This suggests that even individuals with the same BMI can have effects on the prevalence and risk of metabolic syndrome depending on their body image perception. This allows the conclusion that subjective body image perception can function as a supplementary predictor of metabolic syndrome [22]. At the same time, considerations will also have to be made on the strategies to build a correct body image perception.
